# Attitudes, behaviours and strategies towards obesity patients in primary care: A qualitative interview study with general practitioners in Germany

**DOI:** 10.1080/13814788.2021.1898582

**Published:** 2021-03-22

**Authors:** Julian Wangler, Michael Jansky

**Affiliations:** Centre for General and Geriatric Medicine, University Medical Centre Mainz, Mainz, Germany

**Keywords:** Obesity, overweight, general practitioner, primary care, treatment

## Abstract

**Background:**

Obesity poses severe challenges for the health care system. GPs are in an advantageous position to contribute to preventing obesity by diagnosing patients and initiating treatment. Sporadic studies have shown that attitudes towards obesity management in primary care can have a major influence on treating patients successfully.

**Objectives:**

The study focuses on attitudes and behavioural patterns towards obesity patients, willingness to provide care, approaches and strategies, and the challenges experienced.

**Methods:**

After developing the interview guides based on a literature review, 36 GPs in North Rhine-Westphalia and Saarland, Germany, were interviewed between November 2019 and March 2020. Using qualitative typing according to Kluge, different prototypes of GPs were formed. The dimensions of the interview guides were used for deriving the prototypes.

**Results:**

GPs were categorised into four types depending on how they saw themselves and their role in treating patients. The first type (the resigned) was conspicuous through its negative attitude towards obesity management and a lack of willingness to provide care. The second type (the instructors) emphasised the value of active exercise, diet and health promotion, while the third type (the motivators) saw psychosocial support and motivation as a key element in helping patients. In contrast, type four (the educators) focussed primarily on early prevention through patient education.

**Conclusion:**

Depending on which (proto-)type a patient visits, different focuses and strategies are pursued for obesity management and doctor–patient communication. This results in different perspectives and chances of success about therapeutic measures.

KEY MESSAGES

- Many GPs show a lot of sensitivity in treating and supporting obese patients, but different emphases stand out: diet, exercise, psychosocial support, prevention.
- However, some GPs show a sceptical approach.
- Strategies such as motivational consultation and the effort to engage with patients play a central role in long-term treatment outcomes.

## Introduction

Obesity and overweight have been increasing in the European population [1]. Obesity in particular has been gaining focus as a chronic disease with limiting effects on quality of life as well as high levels of morbidity and mortality [2]. In Germany, 53% of adults are currently overweight (BMI 25–29.9 kg/m^2^), of which 17% are obese (BMI >30 kg/m^2^) [1,3]. Weight problems and obesity are increasing in most of the European Union states, with estimates of 52% of the EU’s population (18 and over) overweight in 2014 [1].

Obesity is assumed to play a dominant role in around 80% of type 2 diabetes mellitus cases, 35% of ischaemic heart disease cases, and 50% of hypertensive disease cases in Europe [4]. The negative psychological effects on those affected should not be underestimated either [2,5–7].

Due to their role in primary care, general practitioners treat their patients in a holistic, comprehensive and continuous way to be well aware of the patients’ background. Consequently, they are ideally placed to contribute towards preventing obesity by diagnosing patients and initiating treatment in a timely fashion. They have a variety of options to reduce bodyweight in their patients through a change in lifestyle and positively impact long-term compliance and motivation [8]. These options include consultation on exercise and diet, therapeutic intervention and arranging external healthcare services. Patients requiring additional psychosocial stabilisation may also be referred to psychological intervention. Treatment options using drugs and surgery may also be taken into consideration [1,9].

Studies have shown that obese and overweight patients are more frequently motivated towards losing weight if they consult with their GP about their bodyweight [10,11]. However, there is evidence that only some general practitioners give actual recommendations or instructions on diet and exercise after diagnosing overweight or obesity [12,13].

Severely overweight patients are often dissatisfied with the care given by general practitioners [14]; conversely, European studies have shown broad scepticism amongst GPs regarding motivation and discipline towards sustainable bodyweight reduction in their patients [15–18]. This results in a major shift of responsibility for bodyweight reduction towards patients [19,20]. In support of this analysis, one German-language study found that general practitioners take a more passive role in treating obesity because they see a lack of patient motivation as the greatest hindrance to successful treatment [21]. Qualitative studies from the UK and Portugal indicate insensitive and inconsistent communication from doctors and latent stereotyping with severe impact on the obesity condition [17,22]. GP trainees are also sometimes affected by low confidence regarding obesity management [23].

Other causes for reticence amongst general practitioners towards obesity management include a substantial lack of adequate primary care programmes or funding for nutrition, exercise and drug treatment coverage from Germany’s statutory health insurance funds [1,5].

Mostly in German-speaking countries there is a lack of studies on attitudes towards obesity management and possible explanations for apparent behavioural patterns in primary care [1,21]. This work focuses on attitudes and behavioural patterns towards obesity patients, willingness to provide care and support, approaches and strategies, and the challenges experienced. Suggestions for improved approaches will be derived from the collected data.

## Method

### Concept of the study

Since little is known about attitudes and behavioural patterns amongst general practitioners in treating obese patients there is a need for a broader exploration of this issue. Consequently, a qualitative approach with semi-structured interviews appeared most appropriate. On the one hand, the topic could be researched as impartially as possible for new aspects, and on the other hand, interviews offer GPs the opportunity to present their points of view and experiences in detail.

### Ethics

During this study, no sensitive patient data were gathered or clinical tests performed. All 36 expert interviews with general practitioners were strictly anonymised. However, the authors of the study contacted the Ethics Commission of the State of Rhineland-Palatinate before beginning the study to ensure that it conformed with the medical professional code of conduct. The researchers identified the participants and requested their written consent to participate in the study.

### Recruitment and sampling

The general practitioners interviewed in this study have their practice in the federal states of North Rhine-Westphalia and Saarland, Germany. As part of a qualitative exploratory approach, a limited number of practices in both federal states were contacted, although they were systematically selected. First, a pool of 72 potential contact addresses was set up, including a wide range of general practitioners in both federal states. Subsequently, the recruitment of the sample took place. By using predefined quotas, emphasis was placed on ensuring that certain characteristics are equally represented in the sample (gender, office type, office environment). In addition, attention was paid to a broad geographical distribution of doctors’ offices and the representation of different age groups as well as various qualifications and training backgrounds (Table 1).

**Table 1.** Sociodemographic characteristics of the sample (*N* = 36).

Office type	17 joint offices, 19 single offices
Office environment	12 in small towns or rural communities, 14 in medium-sized towns, 10 in cities
Employment type	24 offices owned by the GP, 12 GPs in employment
Age	Ave. 54 years
Gender	18 male, 18 female
Previous knowledge and qualifications	8 from further training, 7 from additional training in sports medicine, 3 from additional training in psychotherapy or psychoanalysis

A total of 49 physicians were contacted *via* telephone or e-mail, with 36 interviews finally being carried out. The interviews took place between November 2019 and March 2020 and were conducted by two general practice researchers, each conducting half of the interviews. Each interview was carried out either in person or by phone and lasted between 40 and 90 min. Table 1 provides an overview of the participating samples.

### Investigation tools

The interview guides were developed based on a literature review [10,12,14,15,22]. In the course of the first interviews, the instrument was further specified.

The interview guides consist of 24 superordinate questions with several sub-questions and primarily focussed on the following topics: comprised causes of obesity; attitudes towards the condition; identification, patient approach and education; role and self-perception with regard to obese patients; (long-term) therapy support and willingness to treat; preferred approach to obesity management; care and the challenges experienced; subjective assumptions and experiences on efficacy; cooperation with other care services. Personal positions and previous experiences were not included (Supplementary Appendix 1).

### Data analysis

In qualitative research, theoretical saturation is achieved when collecting further data and its analysis do not reveal any new aspects of a category system and, thus, no longer reveal any new findings. This became apparent after 36 interviews.

The analysis was based on qualitative content analysis using MAXQDA software [24]. Types of general practitioners were formed during the analysis; this helped improve the assessment of the differences between the interviewees regarding their self-perception and how they understood their role as well as behavioural patterns in obesity management. In doing so, the empirically founded type formation according to Kluge was applied [24]. A type formation means that an object area is divided into groups or types based on defined characteristics. In terms of content, each type is defined along certain comparative dimensions (indicators) with certain characteristics; so certain common characteristics and properties are within each group.

The type formation procedure takes place in four general stages: Developing relevant comparison dimensions (indicators); grouping of cases and analysis of empirical regularities; analysis of contextual contexts and type formation; characterisation of the derived types.

The central dimensions of the interview guides, self-perception/role and behavioural pattern, were used as indicators for deriving the types. Therefore, they define the within-group similarities and at the same time the between-group differences.

It is important to note that the final types each represent a prototype, meaning a basic pattern in terms of attitudes and behaviour towards obesity patients.

## Results

**Figure 1.**
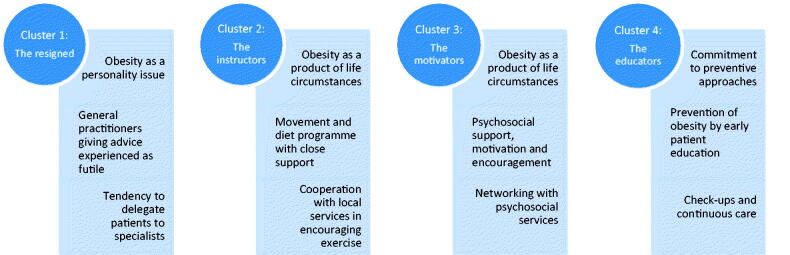
General practitioner prototypes within the sample.

Four different types emerged from the interviews (Figure 1).

The first type (the resigned) was widely represented in the sample and conspicuous through its negative attitude towards obesity management, the patients affected, willingness to provide treatment, and assumptions on the general practitioner’s options in making an improvement.

The other three types showed a more open-minded and proactive approach to dealing with obesity patients, albeit to varying degrees. These general practitioners regarded primary care as an important part of a patient’s journey towards weight reduction. The second type (the instructors) emphasised the value of active exercise, diet and health promotion while the third type (the motivators) saw psychosocial support, stabilisation and motivation as a critical element in helping patients in the long term. In contrast, type four (the educators) focussed primarily on early prevention of severe obesity through patient education.

### Type 1: The resigned

A third of the sample (12) followed a resigned or negative approach with regard to treating obesity patients, owing largely to the negative key experiences they reported. Some of the interviewees went as far as stereotyping, emphasising that obesity was ‘not a disease like any other’, but mainly due to character predisposition involving living to excess or the ‘urge to let themselves go’.

*I’ll say it straight out: It’s often their fault. […] All they do is eat and sit in front of the TV, they never restrain themselves, they can’t be bothered to do anything about their hypertension and so on. […] Everyone has to take responsibility. (I-09-m)*

These general practitioners experienced severely overweight and obese patients as unwilling to take advice, ‘regularly getting nowhere’ initiating a change in attitude and lifestyle, whether through diet or exercise therapy. Instead, the interviewees reported a lack of compliance and many setbacks in therapeutic intervention resulting in long-term severe medical problems and increased mortality risks as well as ‘frustration and jadedness with this type of patient’ on the part of general practitioners.

*Many just won’t listen to medical advice, high-risk patients and walking time bombs. […] What can I do about it? With the best will in the world, you can’t get them interested in making any changes; they don’t have any motivation towards changing anything in their lifestyle. (I-02-m)*

There were indications for communication with obesity patients occasionally turning harsh and disrespectful. There was a recognisable lack of willingness to look for new ways and solutions for the patient to lose weight or advise the patient on the importance of regular exercise and proper diet. By placing the blame for the condition on patient’s individual characteristics, general practitioners of this type also lay the responsibility for weight loss on the patient.

Years of frustrating experience in this group has led them to doubt whether general practitioners can manage this type of patient effectively. Instead, they see a role for specialists using drugs, psychotherapy or possibly surgery as the final option.

### Type 2: The instructors

The second type comprised general practitioners who saw obesity mainly as a combination of life circumstances and predisposition. These GPs took early diagnostics seriously and preferred a structured exercise and diet programme for their patients. They also reported successful outcomes from this treatment.

*I see plenty of exercise and, most importantly, on a regular basis as the correct approach in combination with a healthy high-fibre diet. Patients need to be started on a corresponding programme; that will lead to success sooner or later as long as it is strictly adhered to. (I-31-f)*

One particularly prominent feature: These general practitioners were integrated into a local exercise and health promotion network alongside having informal local contacts to draw on. This especially applies to collaboration with gyms and fitness centres, self-help groups along with diet and health consultants. They saw the possibility of easily arranging reliable sports and health activities for patients as essential in providing adequate support for their obesity patients.

*There isn’t anything you can achieve on your own as a GP. You have to see yourself as part of a structure. This structure takes a lot of personal investment at the beginning. […] You can refer your patients with severe obesity to good local partners without much trouble. (I-08-m)*

These interviewees did not show much interest in continuous patient consultation at close intervals. Instead, they saw a priority in a ‘focussed and concentrated jump-start’ to ‘set the scenes for consistent and gradual weight loss’ in an individually matched motivational exercise programme. Once this phase has been mastered and an increase in exercise and healthy diet has been achieved in the patients’ everyday life, these general practitioners give them more responsibility.

*Once you’ve managed to get the patient to accept their change in lifestyle as a matter of course without relapsing into their old habits, long-term weight reduction will follow. (I-16-f)*

Setting a fixed weight loss target was seen as less important than empowering patients to manage the change and alter their habits. These interviewees also saw health apps as beneficial for motivation and daily routine. The use of drugs and surgical procedures is vehemently rejected, as the interviewees see the risk of yo-yo effects. The only exception should be acute medical emergencies.

### Type 3: The motivators

The third type also rejected the use of drugs and surgical procedures to achieve a substantial weight loss. But unlike ‘the instructors’, these interviewees saw their main task in providing intensive psychosocial support for their patients. In their opinion, motivation and encouragement help develop the gradual realisation amongst patients that it would be beneficial for them to change their lifestyle. The interviewees in this type held the opposite attitude to those in the first type (‘the resigned’).

*Nobody chooses to be fat or feels comfortable about it. This may be a question of predisposition or pre-existing conditions in some individual cases, but in most cases it’s due to longstanding social and psychological processes. Stress at work or adverse life events. […] That’s why it’s so important for the GP to allow the patient to reveal the cause. To help them to help themselves, as it were. (I-17-f)*

These interviewees considered sensitive communication and a collaborative approach to the doctor–patient relationship as important. From their perspective, it is essential to give obese patients enough time for consultation and always remain accessible to them, even when treatment setbacks occur. Three interviewees in this group had undergone additional training in psychotherapy and psychoanalysis and believed that this knowledge played a valuable role in successful long-term obesity management. Like ‘the instructors’ with their involvement in local sports opportunities, networking also played a prominent role in this group – albeit more in the field of psychosocial care and support services. The interviewees also used these services if they believed their obesity patients would benefit from additional assistance.

*Many of these people suffer from depression; they feel uncomfortable with themselves and have low levels of self-esteem and confidence […]. This is where we have to start. […] Not everyone needs psychotherapy. Meeting other people going through the same thing often helps. (I-03-f)*

### Type 4: The educators

This fourth type appears as a variation on the two previous ones. The general practitioners in this group also actively provided care and arrange treatment reasons for their obesity patients. Still, interviewees in this group were far more sceptical as to the success of such treatment compared to the other two groups. They believed that a long-standing case of obesity had ‘already caused damage’ and was ‘not easy to get rid of’. Therefore, they saw it as far more important to deal with the condition earlier on and prevent the development of severe overweight in their patients by pointing out risk factors early, thus bringing about the conditions for a healthy lifestyle. Interviewees in this group took health check-ups as an early warning system very seriously, and some had undergone further training in nutritional medicine.

*Patients on a poor diet with low exercise today are the obesity cases of tomorrow. I think we have one of the health system’s major deficits here. We need doctors that adhere to the prevention aspect as a matter of course without necessarily telling their patients how to live their lives. I mean just this awareness in the population. (I-05-m)*

These interviewees raise the question of how it is possible that patients often only come into the focus of (primary) care after years of obesity.

*If someone’s obese, then something has already gone wrong on the medical side. These people should have been noticed earlier and given the proper care beforehand. So anything that might help us identify these people earlier would be welcome. (I-07-f)*

GPs of this type placed a high value on regular patient contact with consistent education and regular check-ups. Continuous bloodwork would also help identify early risk factors for general practitioners to watch out for. These interviewees also involved their practice staff for support. Here, parts of the practice staff were specially trained on the subject of obesity. In some cases, members of the staff take on tasks in counselling, for example, when it comes to referring patients to further help services or giving advice on healthy eating.

### Additional findings

Respondents from all clusters agreed that successful obesity patient management was often time-consuming, requiring a high level of medical commitment with new attempts at treatment after previous attempts had failed. They also objected to the severe lack of supporting structures and care services for preventing obesity and managing treatment in primary care. There were repeated statements that GPs were often left on their own in caring for and treating obesity patients. They pointed out a lack of informal services for consultation and motivation especially in rural areas. Close-knit networks for dementia had grown in many of Germany’s federal states but there was nothing similar for obesity. In addition, many doctors interviewed openly admitted that they did not have a satisfactory general picture of existing services as there was no fast and straightforward way of navigating through the services available. Apart from that, some interviewees wished for an obesity disease management programme to be initiated with more intense institutionalisation of this disorder since the number of obese patients increases.

## Discussion

### Main findings

The interview results showed a high readiness and sensitivity among most general practitioners treating and supporting overweight and obese patients. Even so, varying strategies and emphases emerged towards stabilising and motivating patients. One group focuses more on early and consistent dietary adjustment and exercise (Type 2: The instructors), while another group concentrates more on psychosocial support (Type 3: The motivators). Especially worth emphasising are the interviewees integrated into informal networks with local gyms and exercise services or psychosocial and behavioural therapists depending on their chosen approach. Another type focuses on preventing severe overweight by pointing out risk factors early, thus bringing about the conditions for a healthy lifestyle (Type 4: The educators).

Although the findings were mainly positive, the interviews revealed a substantial number of GPs showing a sceptic or dismissive attitude towards obesity patients (Type 1: The resigned). This study aimed not to detect any specific stigmatising attitudes, but some of the interviews revealed latent or explicit stereotyping against obesity patients (especially type 1). The lack of readiness to provide care begins with the attribution of low self-discipline and readiness to make lifestyle changes; the cause for overweight is mainly seen in the patient’s personality, such as in lack of willpower.

### Comparison with prior work

This study’s results support general findings from this research field, indicating that obesity is a highly polarising disorder amongst doctors and that the differences in attitudes will lead to differences in the degree of willingness to provide care and treatment [7,22,25]. Previous studies have already noted that general practitioners are often reticent in taking a proactive role in obesity management as they perceive a lack of patient motivation as a serious hindrance [21]. In some cases, pronounced stereotypes and stigmatisations can be observed on the part of doctors [15,16,18]. The present study results point to the work of Teixeira and colleagues, in which the attitudes of Portuguese GPs to obesity management were examined [17]. Here, most doctors expressed the feeling that they are not making any difference in getting their patients to make long-term lifestyle changes; they tend to blame obese patients as unmotivated and not-compliant, very similar to the first type in the present study (the resigned). A Canadian survey of 400 general practitioners comes to similar results [18]. Another commonality between the present study and the work of Teixeira et al. [17] is that GPs feel left alone to a certain extent when caring for obese patients; the desire for more referral options is expressed.

In contrast to the studies mentioned, there is a large part in the present sample (types 2–4) that shows great openness and activity in obesity management. The best practice examples found in the course of the interviews reveal the considerable potential of primary care. They support the widespread assumption that, due to their role as trustworthy and long-term primary care providers, GPs are predestined to care for overweight patients and to positively influence them through therapeutic and communicative measures [10–13]. As Whitlock et al. [8] point out, GPs have a variety of options to reduce bodyweight in their patients through a change in lifestyle and positively impact long-term compliance and motivation.

The present study results show that it ultimately depends on each individual GP to what extent they embrace the clinical picture of obesity and what priorities (instruction, motivation, education) they set to make a difference. A central prerequisite for this is open-mindedness to obese patients and the willingness to collaborate with other organisations and disciplines, just as a mixed-methods study on primary prevention in Germany showed. According to this study, GPs considered (primary) prevention within their realm of responsibility but they saw it ‘as the responsibility of multiple actors in a network of societal and municipal institutions’ [26]. The interviewees of the present study were open to involving other occupational groups for the most part.

Furthermore, the present study results reflect deficits in the structures of obesity care that are perceived by all types. As several studies have shown [12,14,15], there is currently a lack of structured approaches in treating obese patients with continuous support from general practitioners in lifestyle change.

### Strengths and limitations

This interview study revealed a variety of limitations that require further consideration. The GPs interviewed were recruited from a specific region (North Rhine-Westphalia and Saarland, Germany). It is also worth considering that more general practitioners with a specific interest in this topic could have taken part.

A unique feature of the study is that the derivation of prototypes enables a more fundamental overview of attitudes and behavioural patterns of GPs when it comes to obesity management. Yet, the selected method of type formation must be considered self-critically. As already mentioned, the aim here was to compress the data material in order to represent prototypes. At the individual level, there are differences as well as overlaps and common characteristics between the types 2 to 4.

The types formed could be used as a starting point for future research to examine to what extent these basic patterns of attitudes and positions on obesity management exist in everyday practice among general practitioners. In addition to broader quantitative surveys, work with prototypical case vignettes would be conceivable. The use of focus groups could also help to work out these basic patterns based on a controlled discussion. This can already be linked to a preliminary study [23].

### Implications

Some general practitioners tend to see the causes of obesity in the patient’s personality. Therefore, it seems advisable to raise awareness among GPs that obesity can have as complex background involving factors such as life circumstances and pre-existing conditions [1,3,5].

Within the sample, best practice examples can be found for motivational consultation and the effort to engage with patients in their personal situation. Such behavioural treatment strategies can play a central role in long-term treatment outcomes [12].

Some interviewees took early diagnostics seriously and preferred a structured exercise and diet programme. This combination of diagnostics and recommendations is crucial according to existing guidelines, which provide additional assistance [27].

General practitioners should be encouraged in their role as mediators by referring their patients to an extended healthcare network, including psychotherapists or dietary assistants [28]. For example, health authorities often provide a useful guide to the local training and consultation services available.

As suggested by several GPs in the study, developing structured care programmes for obesity management seems sensible. These programmes should aim towards improving patient care and training for GPs and their practice staff. International model projects may provide guidance and could be adapted to suit the specific situation [27–30].

Ideally, general practitioners should be placed in a position where they can fulfil two main tasks – individual consultation and treatment as well as coordination within a multidisciplinary obesity care network [26,28].

## Conclusion

GPs can play an important role in caring for obese patients. This applies to both motivating communication with patients and (therapeutic) measures. The study showed that general practitioners have very different attitudes and experiences about obesity management. These can be classified into four (proto-) types that impact the willingness to provide care and support as well as specific approaches and strategies for obesity management and doctor–patient communication. In terms of therapeutic measures, this results in different perspectives and chances of success.

## Supplementary Material

Supplemental Material

## Data Availability

Research data is available upon request.
