# Supplementary material for: Attitudes, behaviours and strategies towards obesity patients in primary care: A qualitative interview study with general practitioners in Germany
Source: Eur J Gen Pract. 2021 Mar 22;27(1):27–34. doi: 10.1080/13814788.2021.1898582 (PMC7993392; doi:10.1080/13814788.2021.1898582)
Supplement: Supplemental Material [file IGEN_A_1898582_SM4208.docx]

**Interview Guidelines**

- From your experience, what are common causes of obesity? What role do psychosocial problems play?
- How often do you deal with obese patients in everyday practice? Has their number increased in recent years? (Why do you think is that so?)
- Do patients come to you specifically because of their obesity or do they visit your consultation hour for other reasons, so that the topic may come up casually?
- Do patients usually address their obesity on their own or do you usually address that topic? Is there a point at which you start asking patients about their overweight?
- Do you see the general practitioner as the primary contact for the advice and treatment of obesity or should these patients better be referred to specialists (e.g. diabetologist, nutritionist)?
- How often do you advise patients with obesity? How often do you refer patients to a specialist or specific facility for lifestyle, weight or disease management advice? Where do you refer these patients to?
- Do you usually suggest disease management (lifestyle change) or do the patients ask you to do so?
- From your experience, how easy or difficult is it to convince overweight patients to get involved in therapy? How do these patients react when you tell them about their obesity and recommend weight reduction / lifestyle changes? (behavior, motivation, willingness)
- Do you have a specific strategy to motivate patients to change their lifestyle or to get treated? What is particularly important here?
- In your opinion, what are reasonable occasions or opportunities for lifestyle / weight advice in everyday practice? (e.g. check-up)
- What content should lifestyle / weight counseling include?
- In your opinion or experience, what are the most promising strategies for achieving weight loss?
- Do you have specific procedures / strategies in order to promote motivation and compliance? (e.g. acting in partnership, avoiding pressure to succeed, taking into account the patient's ideas)
- Do you refer obesity patients to counseling and care services? (nutritional advice, sport and exercise, psychosocial support, weight watchers, health insurance offers, self-help groups)?
- Would you generally recommend surgical measures to obese patients? Why (not)? Under what circumstances would this be an option for you?
- Do you follow guidelines when advising and treating obese patients? How useful are these?
- What do you think are the main challenges in managing obesity?
- How would you assess the medium- and long-term effects of therapeutic measures? Where do you see the reasons for unsatisfactory results?
- How easy or difficult is it as a family doctor to induce overweight patients to noticeably lose weight and thus be successful in disease management?
- What is your experience with respect to the course and results of obesity therapies? Were you satisfied with the results? How would you describe the compliance and counseling / therapy adherence of obese patients?
- From your experience, how do general practitioners work together with specialists (diabetologists, nutritionists) when it comes to the management of obesity? Where do you see problems and potential for improvement?
- (To what extent) Are you familiar with the care structures specifically for the care and treatment of obesity patients?
- (To what extent) Do you cooperate with other institutions / services / healthcare providers with regard to the care of obese patients?
- In your opinion, what measures would help general practitioners to manage / treat obesity patients better and more effectively? What should be changed or improved?
